# Sex-specific role of galectin-3 in aortic stenosis

**DOI:** 10.1186/s13293-023-00556-1

**Published:** 2023-10-24

**Authors:** Lara Matilla, Ernesto Martín-Núñez, Mattie Garaikoetxea, Adela Navarro, Ibai Tamayo, Amaya Fernández-Celis, Alicia Gainza, Joaquín Fernández-Irigoyen, Enrique Santamaría, Pieter Muntendam, Virginia Álvarez, Rafael Sádaba, Eva Jover, Natalia López-Andrés

**Affiliations:** 1 Cardiovascular Translational Research, Navarrabiomed (Miguel Servet Foundation), Hospital Universitario de Navarra (HUN), Universidad Pública de Navarra (UPNA), IdiSNA, C/Irunlarrea 3., 31008 Pamplona, Spain; 2 Research Methodology Unit, Navarrabiomed, Hospital Universitario de Navarra (HUN), Universidad Pública de Navarra (UPNA), IdiSNA, Pamplona, Spain; 3 Clinical Neuroproteomics Unit, Navarrabiomed, Hospital Universitario de Navarra (HUN), Universidad Pública de Navarra (UPNA), IdiSNA, Pamplona, Spain; 4 G3 Pharmaceuticals, Burlington, MA USA

**Keywords:** Galectin-3, Aortic stenosis, Sex differences, Valve interstitial cell, Inflammation, Calcification, Angiogenesis

## Abstract

**Background:**

Aortic stenosis (AS) is characterized by inflammation, fibrosis, osteogenesis and angiogenesis. Men and women develop these mechanisms differently. Galectin-3 (Gal-3) is a pro-inflammatory and pro-osteogenic lectin in AS. In this work, we aim to analyse a potential sex-differential role of Gal-3 in AS.

**Methods:**

226 patients (61.50% men) with severe AS undergoing surgical aortic valve (AV) replacement were recruited. In AVs, Gal-3 expression and its relationship with inflammatory, osteogenic and angiogenic markers was assessed. Valve interstitial cells (VICs) were primary cultured to perform in vitro experiments.

**Results:**

Proteomic analysis revealed that intracellular Gal-3 was over-expressed in VICs of male AS patients. Gal-3 secretion was also higher in men’s VICs as compared to women’s. In human AVs, Gal-3 protein levels were significantly higher in men, with stronger immunostaining in VICs with myofibroblastic phenotype and valve endothelial cells. Gal-3 levels in AVs were positively correlated with inflammatory markers in both sexes. Gal-3 expression was also positively correlated with osteogenic markers mainly in men AVs, and with angiogenic molecules only in this sex. In vitro*,* Gal-3 treatment induced expression of inflammatory, osteogenic and angiogenic markers in male’s VICs, while it only upregulated inflammatory and osteogenic molecules in women-derived cells. Gal-3 blockade with pharmacological inhibitors (modified citrus pectin and G3P-01) prevented the upregulation of inflammatory, osteogenic and angiogenic molecules.

**Conclusions:**

Gal-3 plays a sex-differential role in the setting of AS, and it could be a new sex-specific therapeutic target controlling pathological features of AS in VICs.

**Graphical Abstract:**

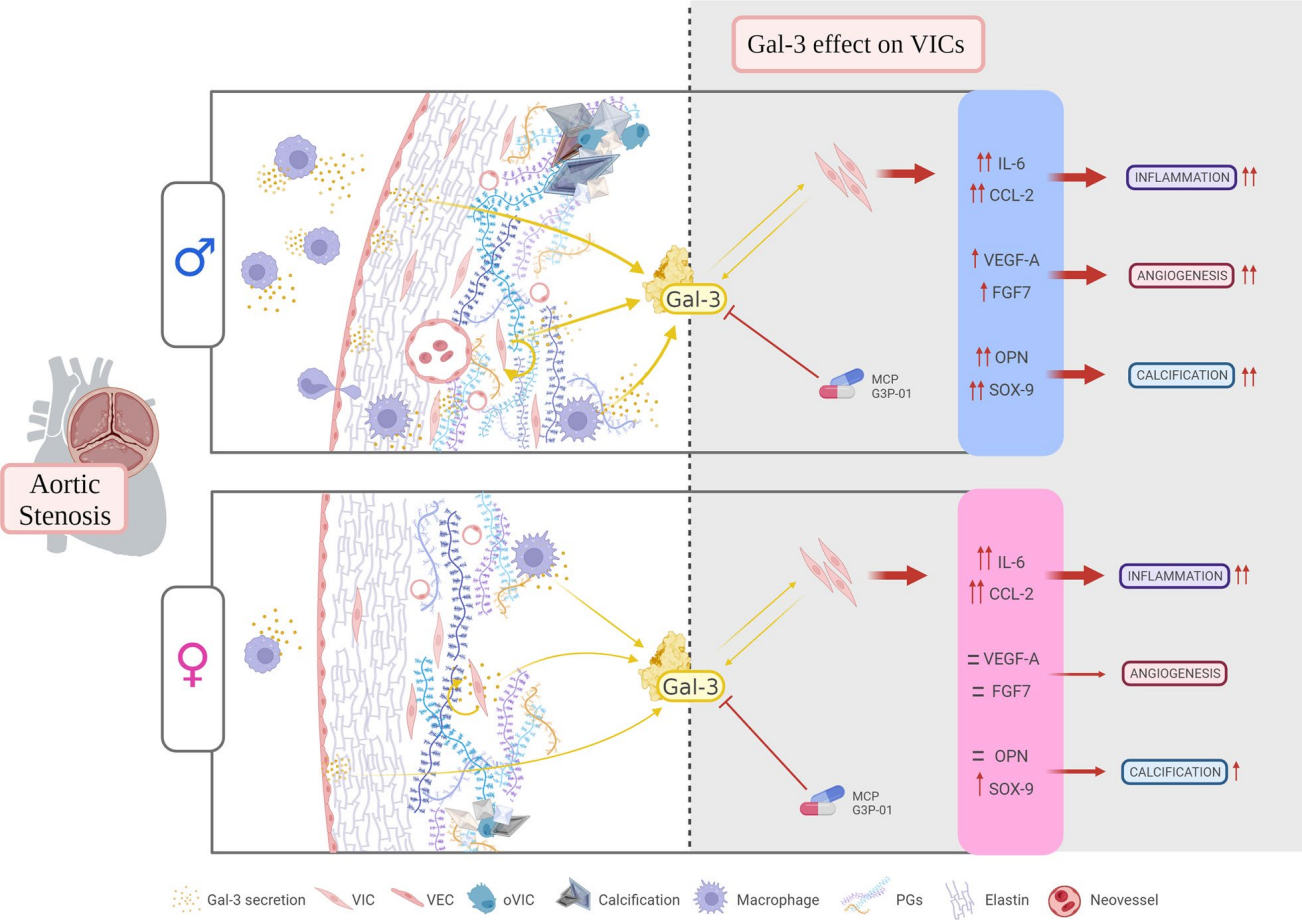

**Supplementary Information:**

The online version contains supplementary material available at 10.1186/s13293-023-00556-1.

## Plain language summary

Aortic stenosis (AS) is a condition that affects the aortic valves (AVs) of the heart and leads to death if untreated. Males and females show clear differences in the onset of AS, both clinically and in valve deterioration. In this study we identified galectin-3 (Gal-3) as a molecule involved in the development of AS alterations with different effects in men and women. We analyzed AVs of 226 patients (139 male and 87 female) with severe AS who underwent surgical AV replacement to study the association of Gal-3 with markers of mechanisms related to AS, such as inflammation, calcification and blood vessels formation. We performed experiments in valvular interstitial cells (VICs) to evaluate the impact of Gal-3 in these cells and its potential use as a therapeutic target. Our results showed that Gal-3 was more expressed in AVs and VICs of men over women. In AVs, Gal-3 levels were associated with inflammatory markers either in male and female, while they correlated with osteogenic markers mainly in men and with angiogenic only in male. The treatment of VICs with Gal-3 produced increased levels of inflammatory and osteogenic molecules by cells of both sexes, but of angiogenic markers only in male’s. Pharmacological inhibition of Gal-3 prevented the increase of these pathological markers in VICs. Overall, our study indicates that Gal-3 is a molecule implicated in the setting of AS in a sex-differential way and its targeting may lead to a new sex-specific therapeutic option for AS treatment.

**Supplementary Information:**

The online version contains supplementary material available at 10.1186/s13293-023-00556-1.

## Highlights

- The existence of a well-known sex dimorphism in the pathophysiological mechanisms of AS, leads to consider a possible differential role for Gal-3 in men and women AVs and VICs and in the pathological features of AS, such as inflammation, angiogenesis and calcification.
- Gal-3 expression in AVs and VICs is significantly higher in male AS patients than in female with the same AS severity.
- Gal-3 is predominantly expressed by activated VICs (vimentin+/α-SMA+) and endothelial cells (CD31+/VE-cadherin+) in men’s AVs, while in women it is mainly expressed by quiescent VICs (vimentin+/α-SMA-) and endothelial cells.
- Gal-3 is directly associated with pathological features of AS in AVs and VICs but in a sex-differential way.
- Targeting Gal-3 inhibition may lead to a new sex-specific therapeutic for AS.

**Supplementary Information:**

The online version contains supplementary material available at 10.1186/s13293-023-00556-1.

## Background

Aortic stenosis (AS) is the most frequent heart valve lesion in Europe and North America with a prevalence of 2–7% in individuals > 65 years. As a consequence of the ageing population, its prevalence is rising promptly and is expected to double in the coming decade [1–3]. The pathogenesis of aortic valve (AV) stenosis is complex and includes features such as inflammation, fibrosis, angiogenesis and calcification [4, 5]. Numerous studies support a sexual dimorphism in AS, both in clinical presentation and patient management, as well as in its pathobiology [6–9]. AVs and valve interstitial cells (VICs) from men exhibit enhanced inflammation, oxidative stress, angiogenesis, apoptosis and calcification compared with women for the same AS severity [8, 10]. Despite advances in research, the only available strategies to improve survival in patients with severe AS are surgical AV replacement and transcatheter AV implantation [3]. Thus, targeted drug therapy to AS progression is still an unmet clinical need.

Galectin-3 (Gal-3) is a β-galactoside binding lectin involved in physiological and pathological processes, including inflammation, fibrosis, angiogenesis and calcification [11–14]. In AVs from AS patients, endogenous expression of Gal-3 is increased and associated with inflammation and osteogenesis [15]. In fact, in vitro treatment with Gal-3 induces inflammatory and fibrotic responses in VICs and modulates their osteogenic differentiation [15, 16]. Blockade of Gal-3 in VICs prevents all these pathological processes, highlighting the critical role it plays in the development of AS [15]. The information about sex differences in Gal-3 remains scarce and controversial. It has been described that circulating levels of Gal-3 are slightly higher in women than in men with heart failure [17], possibly as a consequence of increased body fat presented in women [18]. In the endometrium, 17β-estradiol, progesterone and human chorionic gonadotropin promote the expression and secretion of Gal-3 [19, 20]. However, others have shown lower Gal-3 levels in women than in men with heart failure [21, 22].

The existence of a well-known sex dimorphism in the pathophysiological mechanisms of AS, leads to consider a possible differential role for Gal-3 in men and women. The aim of this work was to study the expression of Gal-3 in AVs and VICs from a cohort of patients with severe AS according to sex, along with its relationship with pathological features of AS, such as inflammation, angiogenesis and calcification.

## Methods

### Clinical cohort

This prospective and observational study included 226 patients with severe AS (AV area ≤ 1 cm^2^ and/or transaortic mean pressure gradient > 40 mmHg) referred to Hospital Universitario de Navarra for surgical AV replacement from June 2013 to November 2021 according to the current guidelines [23]. Other diseases like moderate or severe concomitant valvular disease, malignant tumour, infective endocarditis, diabetes mellitus and chronic inflammatory diseases were elected as exclusion criteria. All patients were evaluated by transthoracic echocardiography. Venous blood was drawn on admission for surgery for the measurement of routine laboratory parameters.

AVs obtained from valve replacement surgery were cut in three, using one third for VIC extraction and culture, another third for protein and RNA extraction, and the last third for histological and immunohistochemistry analyses. Informed consent was obtained from each patient. The study protocol was approved by institutional human research committee (Comité Ético de Experimentación Clínica. Gobierno de Navarra, Departamento de Salud; Ethics numbers 17/2013 and PI2019/59) and it conforms to the ethical guidelines of the 1975 Declaration of Helsinki.

### Cell isolation and culture

Human VICs were isolated from 26 AVs (14 men and 12 women) obtained during surgical AV replacement. VICs from each patient were isolated and individually assayed, as previously described [15]. In brief, AVs were minced and enzymatically digested into 2 mL of buffered-collagenase type 2 (240 U/mg of tissue) for 1 h, and were pelleted by centrifugation. VICs were cultured in DMEM F-12 medium (Gibco) supplemented with 20% fetal bovine serum (FBS) (Gibco), 1% Penicilin/Streptomicin (Lonza), 5 µg/ml insulin (Sigma Aldrich) and 10 ng/ml of fibroblast growth factor (FGF-2) (Novus Biological) at 37 °C and 5% CO_2_ in a saturation humidified incubator (Panasonic) [24]. The VIC phenotype of isolated cells was confirmed at passage 1 by vimentin and alpha-smooth muscle actin (α-SMA) immunocytochemistry. Experiments were performed in serum-starvation conditions (1% FBS) in multiwell plates (Sarstedt). All experiments were carried out in VICs at passage 3–4. At least, four biological replicates (donors) per sex were used in each experiment and 3–8 technical replicates were performed to guarantee the availability of sample to be lately assayed using different techniques. Modified citrus pectin (MCP) and G3P-01 were used as inhibitors of Gal-3. These inhibitors were added in combination with Gal-3 in the cell media at 10^–6^ M and 1 mg/ml respectively, for 24 h.

### SWATH-MS

Sample proteomes were analyzed by Sequential Window Acquisition of all Theoretical fragment ion spectra Mass Spectrometry (SWATH-MS) [25]. Cell pellets were homogenized in a lysis buffer containing 7 M urea, 2 M thiourea, and 50 mM DTT. The homogenates were spun down at 100,000 g for 1 h at 15 °C. Protein quantitation was performed with the Bradford assay kit (Bio-Rad). Protein in-solution digestion, peptide purification, and reconstitution prior to mass spectrometric analysis and library generation were performed as previously reported [26].

MS/MS Library Generation. Peptides recovered from in-gel digestion processing were reconstituted into a final concentration of 0.5 μg/μL of 2% ACN, 0.5% FA, 97.5% Milli-Q-water prior to mass spectrometric analysis. MS/MS data sets for spectral library generation were acquired on a Triple TOF 5600 + mass spectrometer (Sciex, Canada) interfaced to an Eksigent nanoLC ultra 2D pump system (SCIEX, Canada) fitted with a 75 μm ID column (Thermo Scientific 0.075 × 250 mm, particle size 3 μm and pore size 100 Å). Prior to separation, the peptides were concentrated on a C18 precolumn (Thermo Scientific 0.1 × 50 mm, particle size 5 μm and pore size 100 Å). Mobile phases were 100% water 0.1% formic acid (FA) (buffer A) and 100% Acetonitrile 0.1% FA (buffer B). Column gradient was developed in a gradient from 2% B to 40% B in 120 min. Column was equilibrated in 95% B for 10 min and 2% B for 10 min. During all processes, the precolumn was in line with column and flow was maintained all along the gradient at 300 nL/min. Output of the separation column was directly coupled to nanoelectrospray source. MS1 spectra was collected in the range of 350–1250 m/z for 250 ms. The 35 most intense precursors with charge states of 2–5 that exceeded 150 counts per second were selected for fragmentation, rolling collision energy was used for fragmentation, and MS2 spectra were collected in the range of 230–1500 m/z for 100 ms. The precursor ions were dynamically excluded from reselection for 15 s. MS/MS data acquisition was performed using AnalystTF 1.7 (Sciex) and spectra files were processed through ProteinPilot v5.0 search engine (Sciex) using Paragon Algorithm (v.4.0.0.0) [27] for database search. To avoid using the same spectral evidence in more than one protein, the identified proteins were grouped based on MS/MS spectra by the Progroup algorithm, regardless of the peptide sequence assigned. The protein within each group that could explain more spectral data with confidence was depicted as the primary protein of the group. False discovery rate was performed using a nonlinear fitting method [28] and displayed results were those reporting a 1% Global false discovery rate or better.

Quantitative proteomics. For SWATH-MS-based experiments, the instrument (Sciex Triple- TOF 5600 +) was configured as described elsewhere [29]. Briefly, the mass spectrometer was operated using an isolation width of 16 Da (15 Da of optimal ion transmission efficiency and 1 Da for the window overlap), a set of 37 overlapping windows were constructed covering the mass range 450 − 1000 Da. In this way, 1 μL of each sample was loaded onto a trap column (Thermo Scientific 0.1 × 50 mm, particle size 5 μm and pore size 100 Å) and desalted with 0.1% TFA at 3 μL/ min during 10 min. The peptides were loaded onto an analytical column (Thermo Scientific 0.075 × 250 mm, particle size 3 μm and pore size 100 Å) equilibrated in 2% acetonitrile 0.1% FA. Peptide elution was carried out with a linear gradient of 2 to 40% B in 120 min (mobile phases A:100% water 0.1% formic acid (FA) and B: 100% Acetonitrile 0.1% FA) at a flow rate of 300 nL/min. Eluted peptides were infused in the mass spectrometer. The Triple-TOF was operated in swath mode, in which a 0.050 s TOF MS scan from 350 to 1250 m/z was performed, followed by 0.080 s product ion scans from 230 to 1800 m/z on the 37 defined windows (3.05 s/cycle). Collision energy was set to optimum energy for a 2 + ion at the center of each SWATH block with a 15 eV collision energy spread. The mass spectrometer was always operated in high sensitivity mode. The resulting ProteinPilot group file from library generation was loaded into PeakView (v2.1, Sciex) and peaks from SWATH runs were extracted with a peptide confidence threshold of 99% confidence (Unused Score ≥ 1.3) and a false discovery rate (FDR) lower than 1%. For this, the MS/MS spectra of the assigned peptides was extracted by ProteinPilot, and only the proteins that fulfilled the following criteria were validated: (1) peptide mass tolerance lower than 10 ppm, (2) 99% of confidence level in peptide identification, and (3) complete b/ y ions series found in the MS/MS spectrum. Only proteins quantified with at least two unique peptides were considered. The quantitative data obtained by PeakView were analyzed using Perseus software [30] for statistical analysis and data visualization.

### Histology and immunohistochemistry evaluation

Histological determinations in whole AVs were performed in 5 μm-thick paraffin-embedded serial sections using the BOND polymer refine detection systems (DS9800 BOND Polymer Refine Detection and DS9390 BOND Polymer Refine Red Detection) in an automatic immunostainer Leica BOND MAX (all purchased from Leica) following the manufacturer’s instructions. BOND Polymer Refine Detection utilizes a polymerization technology to prepare polymeric HRP-linker antibody conjugates. The detection system is streptavidin and biotin free, thus avoiding non-specific staining as a result of endogenous biotin. All solutions were filled into the bottle-Bond Open Container (Leica) and registered on computer using the Leica Biosystem software. The immunostaining program protocols included: dewax and epitope retrieval solutions, bond wash solutions, peroxide block and incubation steps with 1 or 2 primary antibodies followed by the incubation with HRP (3’-Diaminobenzidine tetrahydrochloride hydrate (DAB)) or AP substrates, as appropriate. In brief, single immunohistochemistry of Gal-3 (mouse, Santa Cruz Biotechnology) was performed using the BOND Polymer Refine Detection (DS9800). After incubating with anti-Gal-3, a post primary rabbit anti mouse IgG followed by a secondary anti-rabbit poly-HRP-IgG were sequentially incubated and the substrate chromogen, 3,3’-Diaminobenzidine tetrahydrochloride hydrate (DAB) was used to visualize the complex via a brown precipitate.

In addition, double immunohistochemistry protocols were conducted for concomitant detection of Gal-3 (Santa Cruz Biotechnology) and the following targets: α-SMA (Sigma-Aldrich), vimentin (Santa Cruz Biotechnology), CD31 (Santa Cruz Biotechnology), VE-cadherin (Santa Cruz Biotechnology), CD68 (Abcam), CD206 (Santa Cruz Biotechnology), CD45 (Santa Cruz Biotechnology), CD80 (Santa Cruz Biotechnology), osteopontin (Santa Cruz Biotechnology), Runx2 (Sigma-Aldrich), Sox9 (Sigma-Aldrich), BMP-2 (Abcam), VEGF-A (Santa Cruz Biotechnology) and VEGFR3 (Santa Cruz Biotechnology). A similar to the above DAB-based protocol was firstly performed followed, when appropriate, by an incubation with a post primary rabbit anti mouse IgG (e.g., when the primary antibody was raised in mouse) and the secondary anti-rabbit poly-HRP-IgG. Thereafter, Gal-3 was detected using the BOND Polymer Refine Red Detection (DS9390). In brief, after incubating Gal-3 (mouse, Santa Cruz Biotechnology), a post primary AP rabbit anti-mouse IgG and a poly-AP anti-rabbit IgG were sequentially incubated and visualized by using an activator and AP substrate included in the kit resulting in a red-coloured precipitate. Incubation with no primary antibody was carried out in negative controls to validate the specific antigen binding and recognition. Histological preparations were imaged using bright field in an automated image analysis system, as appropriate (Nikon). The list of antibodies, working dilutions, catalogue numbers and provider companies can be found as supplementary material (Additional file 2: Table S1).

### ELISA

Gal-3 (R&D System), interleukin (IL)-6, C–C motif chemokine ligand 2 (CCL2), Rantes, ICAM-1, CD44, VEGF-A, FGF-7, VEGFR3, BMP-2, BMP-9, osteopontin and osteocalcin were measured in AVs extracts and cells supernatants according to the manufacturer’s instructions (R&D Systems). For explanted AVs, equal yields of total tissue homogenates (ng/mL or pg/mL as appropriate) were loaded and assayed by ELISA; for in vitro samples, equal volumes of cell supernatants were used and were thereafter normalized by the total protein content (µg/uL) collected from the respective cell monolayers.

### Western blot analysis (WB)

Aliquots of 10–20 µg of total proteins were prepared and electrophoresed from AV and VICs extracts on SDS polyacrylamide gels (4–15% polyacrylamide, Mini-PROTEANTGX Stain-Free, BioRad) and transferred to Hybond-C Extra nitrocellulose membranes (BioRad). Membranes were incubated with primary antibodies for: Gal-3, Sox-9 and CD68; and with secondary antibodies for mouse and rabbit (GE Healthcare). Blot densitometry analyses were performed using Image Lab software. Stain free were used as loading controls for normalization and the net band densitometry was expressed as arbitrary units (AU). Positive blots were detected with a chemiluminiscence method (ECL, Amersham Biosciences) and images acquired with Chemidoc MP Imaging system (Bio-Rad). All western blots were performed at least in triplicate for each experimental condition. Semiquantitative analyses were performed by band densitometry using Image Lab software (Bio-Rad). Alongside the manuscript both the net AU and the fold-change means are discussed for further information. In order to validate the Gal-3 antibody, we performed the knockdown of *LGALS3* gene in VICs using a commercial CRISPR/Cas9‐guided genome editing system to achieve controls of expression, according to the manufacturer's instructions (sc-417680, Santa Cruz Biotechnology, Santa Cruz, CA). Scramble gRNA CRISPR/Cas9 plasmid was used as a control (Additional file 1: Figure S1).

### Statistical analyses

The characteristics of the patients were summarized using frequencies and percentages, means and standard deviations (SD), as appropriate. Data normality was assessed through Shapiro–Wilk’s test and Kolmogorov–Smirnov (with Lilliefors p value). Quantitative variables were analysed by T student test or Mann–Whitney U test if the normality was not met. The effects of sex on Gal-3 levels were assessed in two steps. First, univariate lineal regressions models were fitted for all continuous variables. Similarly, univariate logistic regression models were used to estimate the odds ratios of categorical variables. In a second step, the adjusted effect of sex over the selected variables were analysed using lineal and logistic multivariate regression models. Based on the magnitude of the effect and the p values calculated in the univariate models, age, statin use and creatinine levels were used as covariates of the multivariate models to calculate odds ratio (OR). The absence of multicollinearity was guaranteed making use of the Variance Inflation Factor for each independent variable. Pearson or Spearman correlation coefficients were calculated as appropriate. A *p* value of < 0.05 was considered statistically significant. All analyses in the clinical cohort were performed using the R statistical package, v. 3.6 (R Foundation for Statistical Computing. Vienna, Austria). In vitro, multiple variables were compared using a 1-way ANOVA and a Tukey’s post hoc test or Kruskal–Wallis followed by Dunn’s post hoc test (when normality was or was not met, respectively. GraphPad Software Inc. was used for in vitro analyses.

## Results

### Clinical data in AS patients

The baseline clinical and demographic characteristics of AS patients (median [IQR] age: 72 [66–78] years, 61.50% men) recruited for this study are shown in Table 1. As expected, women were older than men and presented lower height and weight. Whereas the use of diuretics was significantly higher in women (48.2% vs 63.2%, *p* = 0.026), statin intake was higher in men (71.9% vs 47.1%, *p* < 0.0001). Moreover, creatinine was lower in women as compared to men (*p* < 0.001).

**Table 1:** Demographic and clinical data of AS patients

Variables	Total	Men	Women	*p*-value
*n* (%)	226	139 (61.50)	87 (38.50)	n/a
Age (median [IQR])	72 [66–78]	70 [65–77]	77 [69–80]	0.000
BS, cm^2^ (mean ± SD)	1.81 ± 0.20	1.90 ± 0.16	1.67 ± 0.16	0.000
HTA, *n* (%)	156 (69.0)	97 (69.8)	59 (67.8)	0.756
Renal insuficiency, *n* (%)	14 (6.2)	7 (5)	7 (8)	0.361
Creatinine (mg/dL)	0.86 [0.76–1.01]	0.91 [0.81–1.1]	0.79 [0.70–0.94]	0.000
NYHA class, *n* (%)				
I	24 (10.6)	19 (13.7)	5 (5.7)	0.076
II	137 (60.6)	91 (65.5)	46 (52.9)	0.069
III	61 (27.0)	28 (20.1)	33 (37.9)	0.005
IV	4 (1.8)	1 (0.7)	3 (3.4)	0.160
Mean gradient (mmHg)	50.2 (13.6)	49.7 (13.6)	50.9 (13.7)	0.559
Max gradient (mmHg)	78.8 (19.6)	78.6 (19.7)	79.1 (19.7)	0.879
Drug medicines				
ACEi, *n* (%)	58 (25.7)	36 (25.9)	22 (25.3)	0.918
ARB, *n* (%)	54 (23.9)	34 (24.5)	20 (23)	0.801
Diuretics, *n* (%)	122 (54)	67 (48.2)	55 (63.2)	0.026
β-blockers, *n* (%)	65 (28.8)	41 (23.5)	24 (27.6)	0.758
Statins, *n* (%)	141 (62.4)	100 (71.9)	41 (47.1)	0.000

*n/a* no applicable, *IQR* interquartile range, *SD* standard deviation, *BS* body surface, *HTA* arterial hypertension, *NYHA* New York Heart Association class, *eGFR* estimated glomerular filtration rate, *ACEi* Angiotensin-converting enzyme inhibitor, *ARB* Angiotensin II receptor blocker

### Gal-3 expression is up-regulated in male VICs and AVs

**Fig. 1:**
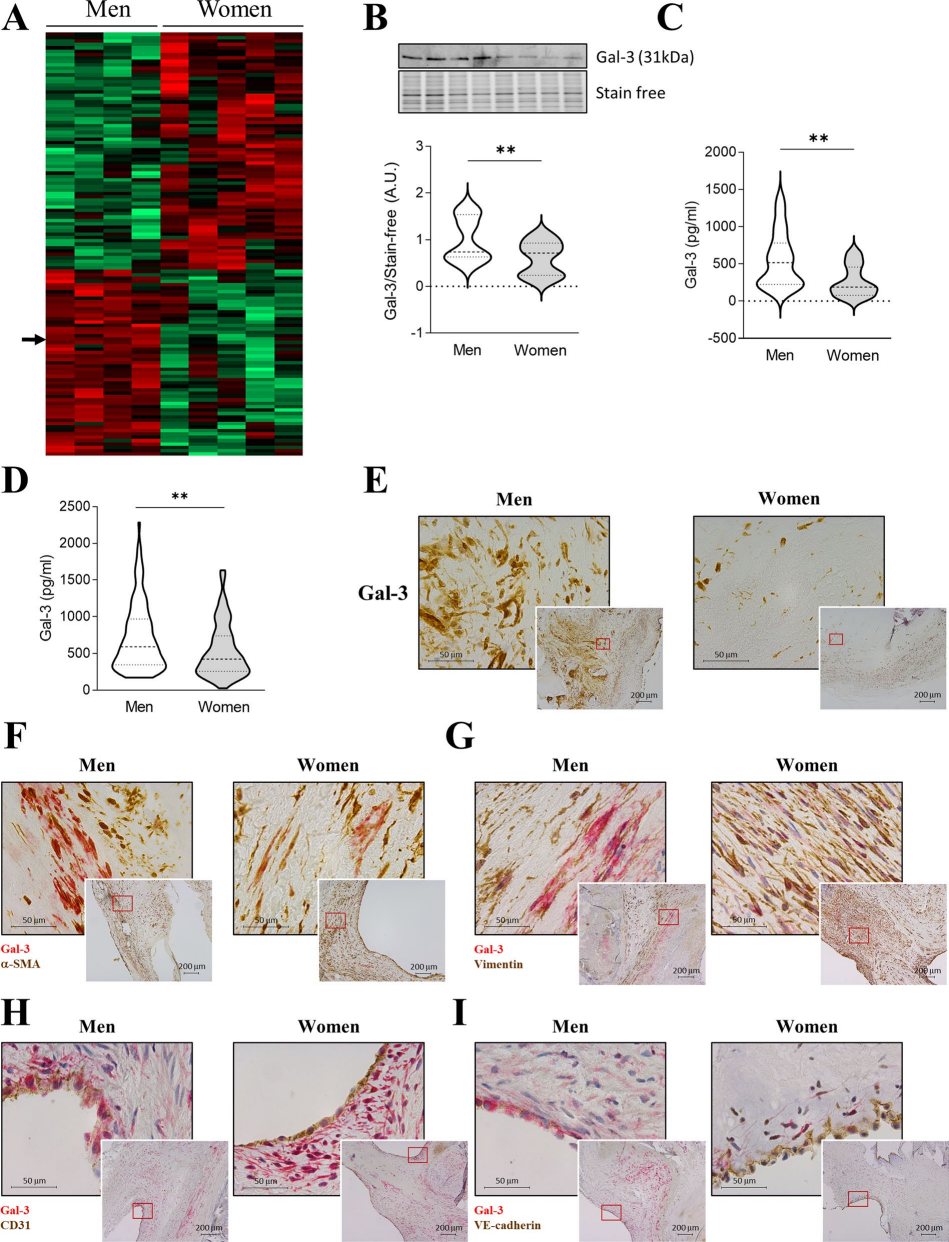
Gal-3 is up-regulated in male VICs and AVs. Proteomic heatmap representing in red up-regulated proteins in men vs. women-derived VICs and in green down-regulated proteins (**A**). Levels of Gal-3 validated in VICs isolated from men and women AVs measured by WB (**B**) and ELISA (**C**). Levels of Gal-3 validated by ELISA in human AVs samples from males compared to females (**D**). Representative microphotographs immunostained for Gal-3 in AVs from men and women (**E**). Representative microphotographs double immunostained for Gal-3 and α-SMA (**F**), vimentin (**G**), CD31 (**H**) and VE-cadherin (**I**). *Gal-3* galectin-3, *CD31* cluster differentiation 31, *SMA* smooth muscle actin. *N* = 108 for men and *N* = 83 for women for AVs and *N* = 20 for men and *N* = 24 for women for VICs. *p < 0.05 vs men, **p < 0.01 vs men

The total number of identified proteins in VICs using SWATH-MS was 1936. Among them, 125 were differentially regulated in male VICs when compared with female VICs; 55 up-regulated in male and 70 down-regulated. Gal-3 was identified as one of the up-regulated proteins in male from AS patients (44.2%, *p* = 0.0102). In Fig. 1A, heatmap representing the fold-change of identified proteins with associated p-values from the pair-wise quantitative comparisons. Significantly up-regulated proteins between pair-wise comparisons are labeled in red and significantly down-regulated proteins in green. Gal-3 up-regulation accounting in men was further validated in VICs isolated from independent AS donors (Fig. 1B). Moreover, Gal-3 secretion was higher in male VICs supernatant as compared to females (528 ± 375 vs. 263 ± 206 pg/ml, *p* = 0.0087) (Fig. 1C). We next tested the protein expression of Gal-3 in human AVs from both sexes by ELISA. Gal-3 was over-expressed in AV tissue from males as compared to females (721.9 ± 455 vs. 541.4 ± 346 pg/ml, *p* = 0.0022) (Fig. 1D). Importantly, men AVs exhibited higher Gal-3 levels (OR = −152.07, *p* = 0.032) after adjusting for age, statins treatment and creatinine. Accordingly, Gal-3 immunostaining was greater in AVs from men, compared to women’s (Fig. 1E).

We have previously reported that Gal-3 was expressed by VICs in AVs from AS patients [15]. We now investigated whether cell types that express Gal-3 could differ between sexes in AVs from AS patients. Double immunohistochemistry evidenced concomitant expression of Gal-3 with α -SMA and vimentin (Fig. 1F-G), being the co-expression greater in men for α -SMA and in women for vimentin. Moreover, Gal-3 was also expressed in valve endothelial cells (VECs) in both sexes as evidenced by the co-localization with CD31 and VE-cadherin, although its expression was higher in the endothelium of men (Fig. 1H–I).

### Gal-3 is associated with inflammation in men and women AVs

**Fig. 2:**
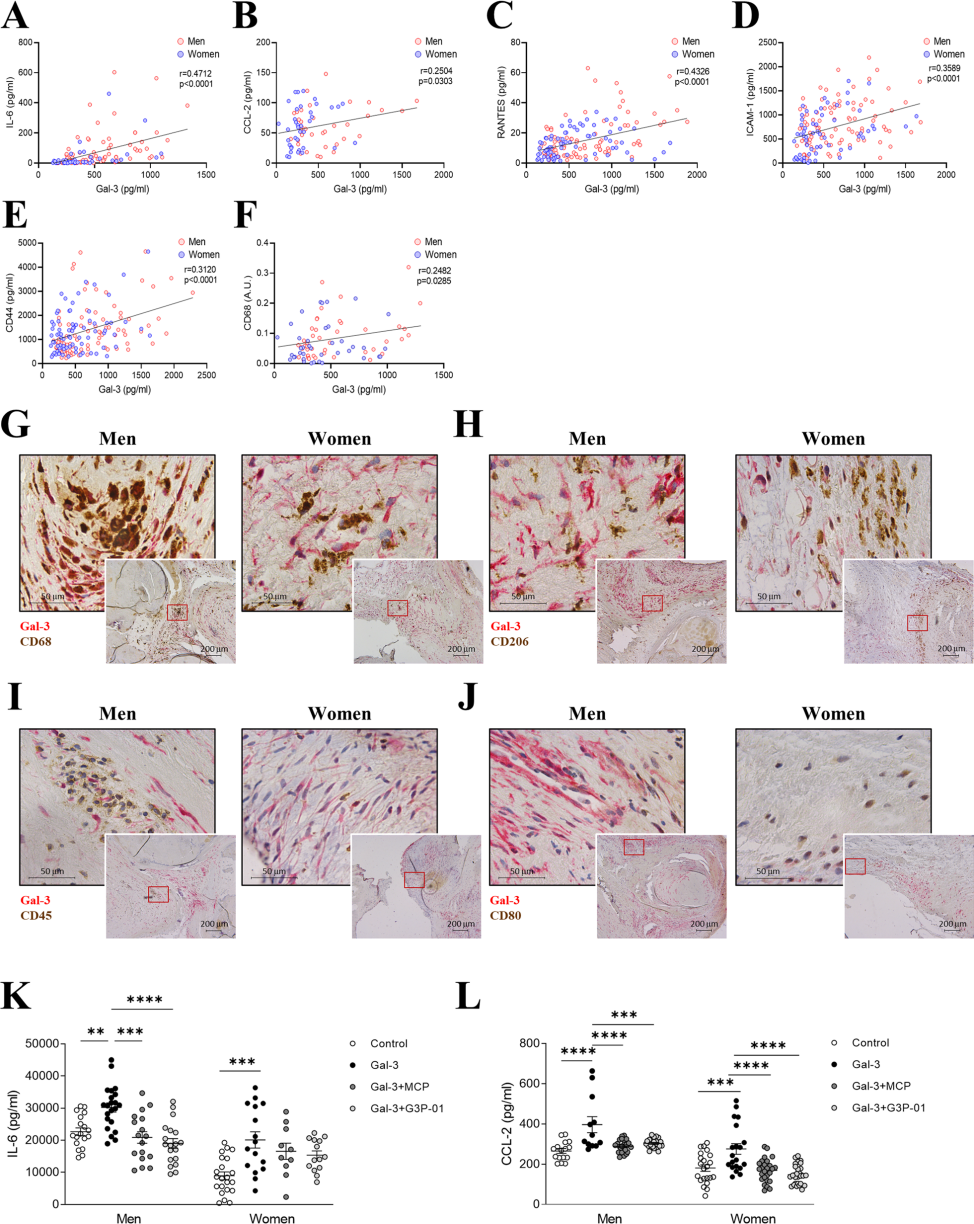
Gal-3 is associated with inflammation in men and women AVs. Positive correlations between Gal-3 and IL-6 (**A**), CCL-2 (**B**), RANTES (**C**), ICAM-1 (**D**), CD44 (**E**) and CD68 (**F**) in AVs from AS patients. Representative microphotographs of AV sections from AS patients immunostained for Gal-3 along with CD68 (**G**), CD206 (**H**), CD45 (**I**) and CD80 (**J**). Protein levels of IL-6 (**K**) and CCL-2 (**L**) measured by ELISA after stimulation in vitro with Gal-3 and with MCP or G3P-01. *IL* interleukin, *CCL-2* C–C motif chemolike ligand 2, *ICAM-1* Intercellular Adhesion Molecule 1, *CD* cluster of differentiation, *Gal-3* galectin-3. *N* = 108 AVs from men and *N* = 83 AVs from women. In vitro: number of biological replicates: 12 men and 12 women; number of technical replicates: 3–6

In Fig. 2, levels of Gal-3 in AVs from the whole cohort were positively correlated with inflammation markers such as IL-6 (*r* = 0.4712, *p* < 0.0001), CCL-2 (*r* = 0.2504, *p* = 0.0303), Rantes (*r* = 0.4326, *p* < 0.0001), ICAM-1 (*r* = 0.3589, *p* < 0.0001), CD44 (*r* = 0.3120, *p* < 0.0001) and CD68 (*r* = 0.2482, *p* = 0.0285) (Fig. 2A-F). Interestingly, these positive correlations were maintained in men and women AVs when analyzed separately (Additional file 1: Figure S2A–J). However, the correlation between Gal-3 and CD68 was present only in men AVs (*r* = 0.3661, *p* = 0.0134, Additional file 1: Figure S1K). Remarkably, the co-expression of Gal-3 with CD68 positive cells was more evident in AVs from men than women (Fig. 2G). Of note, Gal-3 does not seem to co-localize with CD206, CD45, or CD80 positive infiltrates (Fig. 2H–J).

AV-derived VICs were isolated from both sexes with AS and treated with recombinant Gal-3 as previously reported [15] in the presence of two specific inhibitors, MCP and G3P-01 [31]. Stimulation with Gal-3 significantly increased IL-6 secretion in VICs isolated from male (133%, *p* = 0.0027) and female (226%, *p* = 0.001) AS patients (Fig. 2K). The employment of either MCP or G3P-01 blocked IL-6 increase induced by Gal-3 in both sexes VICs (Fig. 2K). Similarly, treatment with recombinant Gal-3 enhanced CCL-2 secretion in VICs isolated from men (149%, *p* < 0.0001) and women (152%, *p* = 0.0007) (Fig. 2L). Gal-3 pharmacological inhibition blunted its effects in cells from both sexes (Fig. 2L).

### Gal-3 is differentially associated with osteogenic markers in men and women

**Fig. 3:**
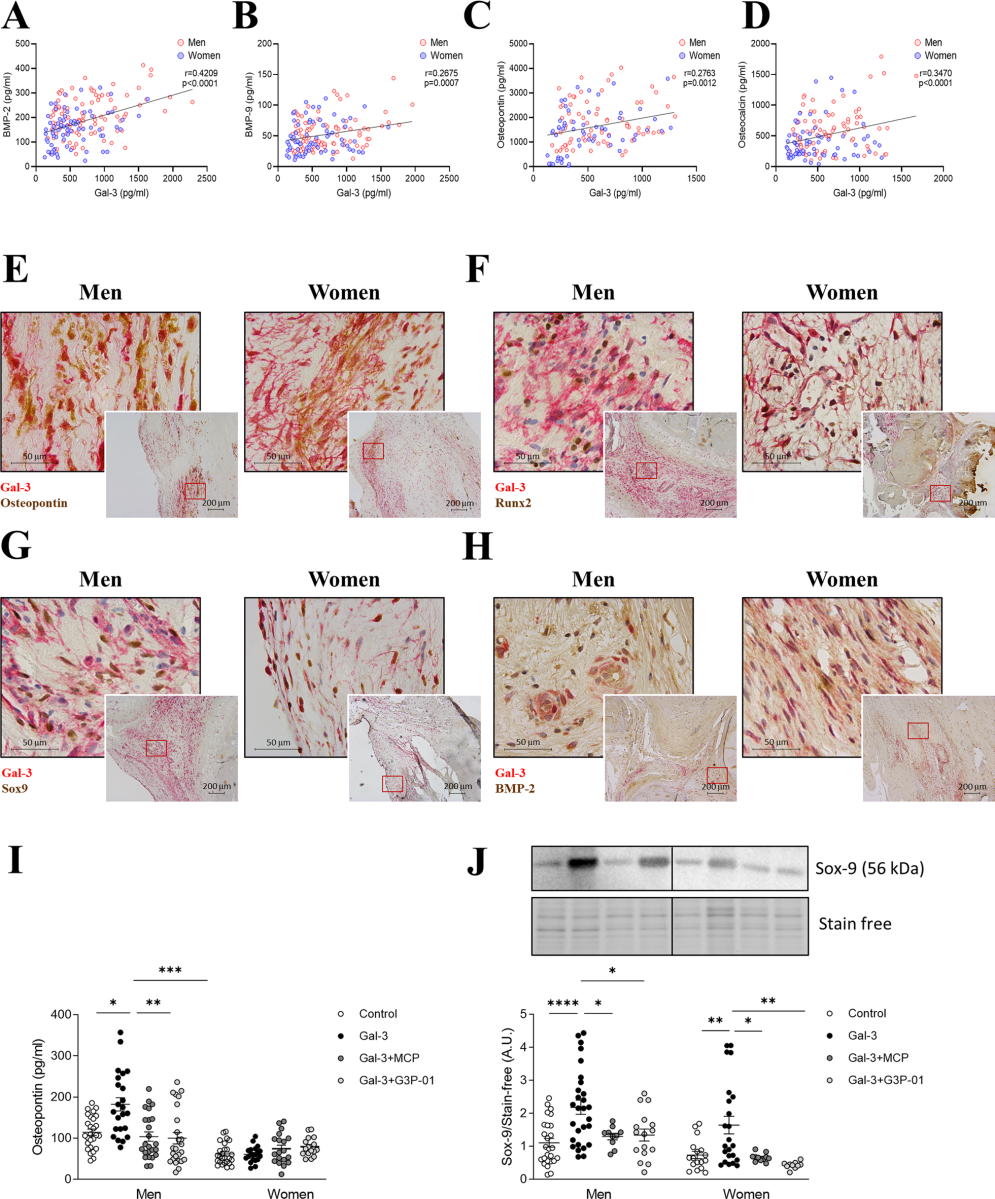
Gal-3 sex-differential effects on osteogenic markers. Positive correlations between Gal-3 and BMP-2 (**A**), BMP-9 (**B**), osteopontin (**C**) and osteocalcin (**D**) in AVs from men and women. Representative microphotographs of AV sections from AS patients immunostained for Gal-3 in combination with osteopontin (**E**), Runx2 (**F**), Sox-9 (**G**) and BMP-2 (**H**). Protein levels of osteopontin (**I**) and Sox-9 (**J**) measured by ELISA and WB respectively, after stimulation in vitro with Gal-3 and with MCP or G3P-01. *BMP* bone morphogenetic protein, *Gal-3* galectin-3, *Runx2* Runt-related transcription factor 2, *Sox-9* SRY-Box Transcription Factor 9. *N* = 108 AVs from men and *N* = 83 AVs from women. In vitro: number of biological replicates: 12 men and 12 women; number of technical replicates: 3–6

Gal-3 has been previously associated with calcification in the AV [15], although the sex-specific pattern has not been investigated yet. As expected, Gal-3 levels positively correlated with the osteogenic markers BMP-2 (*r* = 0.4209, *p* < 0.0001), BMP-9 (*r* = 0.2675, *p* = 0.0007), osteopontin (*r* = 0.2763, *p* = 0.0012) and osteocalcin (*r* = 0.3470, *p* < 0.0001) in AS patients (Fig. 3A–D). All these correlations were found in men AVs (Additional file 1: Figure S3A, C, D, E). In AVs obtained from women, Gal-3 was only correlated with BMP-2 levels (*r* = 0.2770, *p* = 0.0141, Additional file 1: Figure S3B). At the histological level, double immunostaining revealed that Gal-3 was co-expressed with osteopontin, Runx2, Sox-9 and BMP-2 in AVs from both men and women (Fig. 3E–H).

Interestingly, isolated male VICs treated with Gal-3 exhibited increased levels of osteopontin (159%, *p* = 0.0364), effect blunted by its pharmacological inhibition with MCP or G3P-01 (Fig. 3I). Gal-3 did not increase osteopontin secretion in female VICs (Fig. 3I). Nevertheless, Gal-3 stimulation led to increased Sox-9 expression in male (197%, *p* < 0.0001) and female (223%, *p* = 0.0063) VICs, being these effects blocked by MCP or G3P-01 (Fig. 3J).

### Gal-3 exerts pro-angiogenic effects only in men

**Fig. 4:**
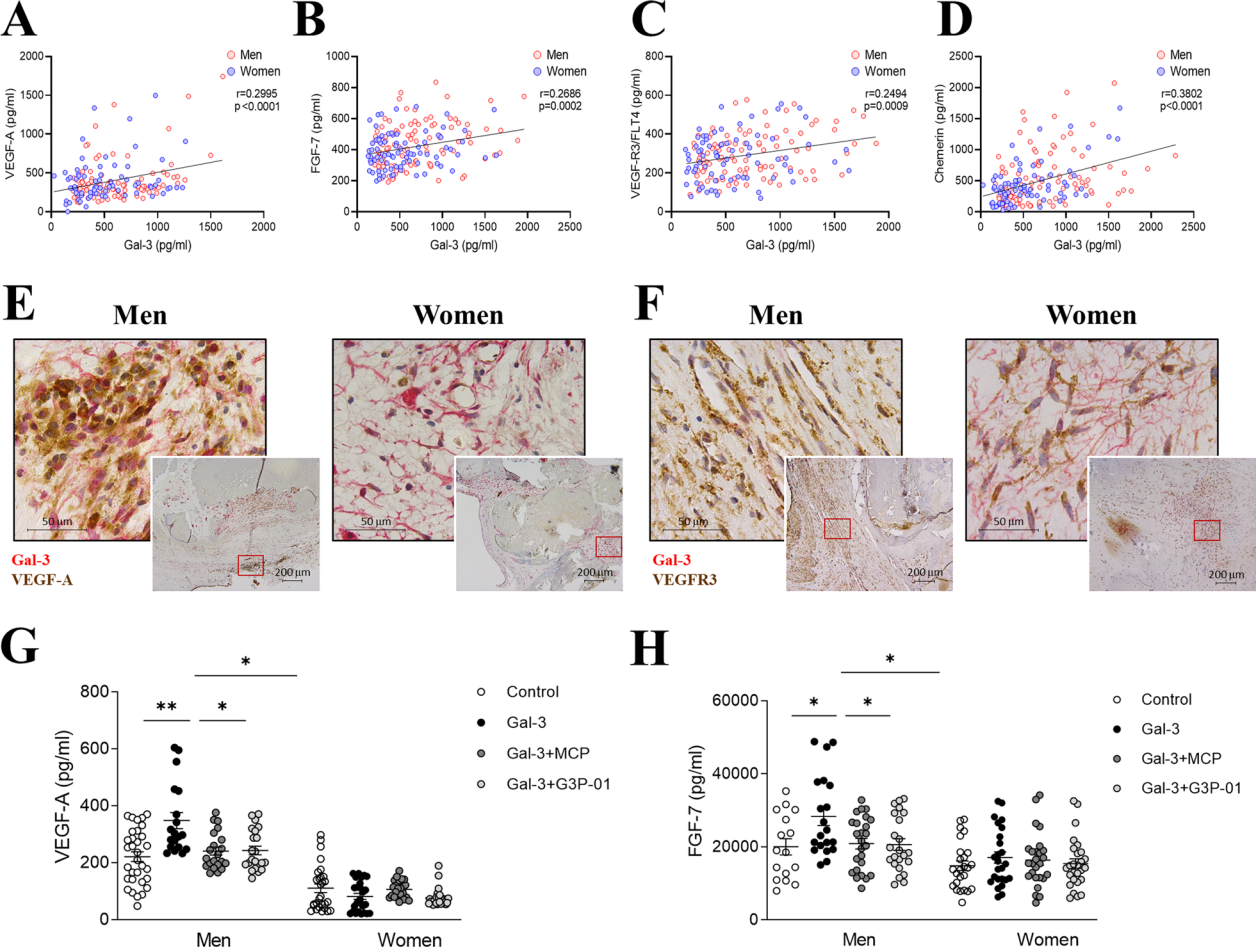
Gal-3 exerts pro-angiogenic effects only in men. Positive correlations between Gal-3 and VEGF-A (**A**), FGF-7 (**B**), VEGFR3 (**C**) and Chemerin (**D**) in AVs from men and women. Representative microphotographs of AV sections from AS patients immunostained for Gal-3 in combination with VEGF-A (**E**) and VEGFR3 (**F**). Protein levels of VEGF-A (**G**) and FGF-7 (**H**) measured by ELISA after stimulation in vitro with Gal-3 and with MCP or G3P-01. *Gal-3* galectin-3, *VEGF* vascular endothelial growth factor, *FGF-7* fibroblast growth factor-7, *VEGFR* receptor of vascular endothelial growth factor. *N* = 108 AVs from men and *N* = 83 AVs from women. In vitro: number of biological replicates: 12 men and 12 women; number of technical replicates: 3–6

We next investigated the possible correlations between protein levels of Gal-3 and markers assessing angiogenesis and lymphangiogenesis, novel key sex-differential processes involved in AS [10]. Gal-3 expression in AVs was positively correlated with pro-angiogenic VEGF-A (*r* = 0.2995, *p* < 0.0001), FGF-7 (*r* = 0.2686, *p* = 0.0002), lymphangiogenic VEGFR3 (*r* = 0.2494, *p* = 0.0009) and chemerin (*r* = 0.3802, *p* < 0.0001) (Fig. 4A–D). In a sex-stratified analysis, the associations between Gal-3 and these angiogenesis markers were only maintained in men (Additional file 1: Figure S4). Immunohistological analyses showed the co-expression of Gal-3 and VEGF-A mainly in men AVs, whereas in women’s AVs co-localization of Gal-3 and VEGF-A was restrained to neovessels (Fig. 4E). However, Gal-3 and VEGFR3 were co-expressed in AVs from both sexes (Fig. 4F).

In vitro, Gal-3 treatment induced VEGF-A secretion only in male VICs (157%, *p* = 0.002, Fig. 4G), but not in women-derived VICs. Similarly, Gal-3 stimulation enhanced FGF-7 secretion specifically in VICs isolated from men donors (141%, *p* = 0.028, Fig. 4H). The use of either MCP or G3P-01 abolished all the above effects (Fig. 4G–H).

## Discussion

The development of effective drug therapies for AS remains an unmet clinical need and sex-specific differences in heart valve diseases need to be investigated. Our study demonstrates for the first time that there is a sex-specific profile for Gal-3 expression in AS, as well as a sexual dimorphism in its role on pathological features of AS. Gal-3 expression is higher in AVs and VICs isolated from AS male donors as compared to women’s with the same AS severity. Interestingly, in men AVs, Gal-3 is predominantly expressed by activated VICs (α-SMA +) and endothelial cells (CD31 + /VE-cadherin +), whilst only in quiescent VICs (α-SMA -) and endothelial cells in women AVs. In AVs, Gal-3 levels positively associate with inflammatory markers in both sexes, while correlating directly with osteogenesis and angiogenesis mainly in men. In VICs, treatment with exogenous Gal-3 induces the expression of inflammation, osteogenic and angiogenic markers in male VICs. Overall inflammation was regulated by Gal-3 in VICs from women. Importantly, pharmacological blockade of Gal-3 exerts beneficial effects against the pathological mechanisms activated within each sex. Thus, Gal-3 could be a new sex-specific therapeutic target controlling these pathological features of AS.

The degeneration of AVs in AS involves structural damage such as inflammation, apoptosis, aberrant extracellular matrix remodelling (ECM), mineralization and neoangiogenesis, events associated with differentiation of VICs into myofibroblast or osteoblast-like cells [4, 5]. Sex dimorphism exists in these AS pathological features [8, 9, 32, 33]. Indeed, male AVs with AS exhibit higher inflammation, oxidative stress, apoptosis, calcification and angiogenesis than female AVs. Moreover, women’s AVs develop enhanced ECM in AS [8, 10]. In porcine VICs, these findings have been confirmed with increased markers of inflammation, calcification and angiogenesis in male cells as compared to female’s [34, 35]. These results have been now expanded to human VICs [8, 10]. Gal-3 has been shown to be an activator of inflammatory, fibrotic and osteogenic responses in the context of heart failure or atherosclerosis [11, 36–40]. Recently, our group have described these same effects of Gal-3 in AS [15], although its role between sexes has not been elucidated [15]. We extend these findings by demonstrating that Gal-3 expression and function is associated with the activation of sex-specific mechanisms underlying the development of AS. Given the higher inflammation and calcification found in men’s AV and VICs, it is plausible to speculate that Gal-3 levels could be increased in men’s as compared to women’s AVs. In this study, we demonstrated using a proteomic approach that Gal-3 was one of the main up-regulated proteins in men-derived VICs as compared to female’s. This was confirmed by WB and ELISA, where we observed higher levels of the protein in the cell extract and supernatant of male-derived VICs. Accordingly, Gal-3 levels were increased in men’s AVs as compared to women’s. Of interest, the source of Gal-3 seemed to be different between sexes, while in women AVs was prevalent in vimentin positive cells, in men’s AVs was predominantly expressed by activated VICs positive for α-SMA (myofibroblastic phenotype). This VIC phenotype is commonly increased in AV pathology [41] and could mark the new transformed mesenchymal cells as a result of endothelial-mesenchymal transition [42]. Moreover, VICs myofibroblastic phenotype could precede osteoblast phenotype [43], implying a very early Gal-3 role in osteogenic differentiation within the male’s valve. Furthermore, Gal-3 was expressed in endothelial cells of both sexes but enhanced in men’s AVs, suggesting that it could also play a role in their transformation into mesenchymal cells as it has been described for other pathologies [44–46]. The clinical impact of a higher number of cell types acting as a source of Gal-3 within the male AV may potentially impact the pathological burden between sexes. Indeed, exogenous Gal-3 elicited multiple pathological mechanisms contributing to AS, while overall inflammation was triggered in female VICs. Future studies are warranted to study Gal-3 effects on valve endothelial cells and its possible interaction with sex.

Gal-3 has been involved in cardiovascular inflammation [47] and calcification [13, 16, 48, 49]. There are also some evidences suggesting that Gal-3 could have pro-angiogenic properties in human umbilical vein endothelial cells and in cancer [12, 50]. Nevertheless, the role for Gal-3 in VICs has hardly been explored [15, 16]. Our results presented here show sex similarities and differences in Gal-3 effects in VICs-derived from men and women. Gal-3 associated to inflammation in AVs from men and women. Accordingly, VICs responded to Gal-3 by secreting pro-inflammatory molecules in cells isolated from both sexes. Moreover, Gal-3 levels also associated with calcification markers, predominantly in men’s AVs, although BMP-2 association was also presented in women’s AVs. Consequently, Gal-3 induced the master transcription factor for osteochondrogenic differentiation Sox-9 in VICs from both sexes, although the induction was higher in male-derived VICs. Of note, Sox-9 well known marker for the study of ectopic cardiovascular calcification [51]. Neovascularization may precede or promote inflammation [52, 53] and perpetuates osteogenesis in advanced calcified AVs [54]. In our study, we show that Gal-3 could exert pro-angiogenic effects but only in male VICs. These results were reinforced at the ex vivo level, showing the positive association between Gal-3 expression and pro-angiogenic markers only in men’s AVs. Thus, the results presented here suggest that the greater presence of Gal-3 in male AVs and its sex-differential effects in VICs could be responsible, at least in part, for the increased calcification and angiogenesis in male valves relative to women already reported in AVs from AS patients [8, 10].

Gal-3 blockade could be a promising therapy in the context of AS. Either by gene knock-down or by pharmacological inhibition, blocking Gal-3 reduces inflammation, fibrosis and osteogenesis in VICs [15] or VEGF-A mediated angiogenesis [55]. In agreement with these data, the use of the Gal-3 inhibitors MCP or G3P-01 prevented VICs from Gal-3-induced secretion of pathologic targets within each sex.

This study had several limitations. First, we have not explored the role of Gal-3 in VECs, another important cellular element in the pathophysiology of AS. Future in vitro studies analysing Gal-3 effects on VECs and the interactions between VICs and VECs would be appropriate to parallel findings in end-stage clinical samples. Second, AS patients had been treated with antihypertensive drugs or statins which could modify Gal-3 levels [56]. Nevertheless, the only sex-difference was found for statin intake, which was higher in men than in women. Third, although most of AS patients included in the study had preserved renal function, we found significant differences in eGFR between men and women. Since high Gal-3 levels are related to impaired renal function, it is important to consider this factor. However, our results indicate that there is indeed a sexual dimorphism in Gal-3 levels, since men presented higher expression of this molecule and a more conserved eGFR.

### Perspectives and significance

In summary, we report a sex-specific association for Gal-3 with biomarkers of pathophysiological mechanisms of AS, i.e. inflammation, calcification and angiogenesis. Herein, Gal-3 expression in AVs and VICs was significantly higher in male AS patients than in female with the same AS severity. Interestingly, Gal-3 was predominantly expressed by activated VICs (vimentin + /α-SMA +) and endothelial cells (CD31 + /VE-cadherin +) in men’s AVs, while in women it was mainly expressed by quiescent VICs (vimentin + /α-SMA-) and endothelial cells. Our study highlights that Gal-3 is directly associated with markers of AS pathological features in AVs and VICs in a sex-differential way, suggesting that Gal-3 inhibition may lead to a new sex-specific therapeutic option for AS. Future directions include deepen into the mechanisms that explain Gal-3 sex-differential expression in stenotic AVs. In addition, in-depth mechanistic experiments dissecting the potential role of Gal-3 and its inhibition in inflammatory, angiogenic and osteogenic pathways in valvular cells will be mandatory. Finally, specific sex-based interventions (e.g., hormones) should be considered as potential options to modulate Gal-3 expression in the setting of AS.

## Conclusions

In conclusion, Gal-3 emerges as a new therapeutic target in AS, with important sex-specific implications. Gal-3 pharmacological inhibition could protect from AV inflammation, calcification and angiogenesis in men’s AVs, whereas Gal-3 overall prevent inflammation and does not seem to play a role in angiogenesis in women’s AVs.

### Supplementary Information

**Additional file 1**: **Figure S1**. Gal-3 antibody validation in *LGALS3-*knockdown VICs. Immunoblot for Gal-3 commercial antibody in CRISPR/Cas9 edited VICs for scramble control (left) or *LGALS3* gene knock-down (right) (A). Stain-free gel for the Gal-3 antibody validation immunoblot (B). **Figure S2.** Gal-3 correlates with inflammation markers both in men and women AVs. Positive correlations between Gal-3 and IL-6 (A-B), CCL-2 (C-D), RANTES (E–F), ICAM-1 (G-H), CD44 (I-J) for both men and women AVs. Positive correlation Gal-3-CD68 (K-L) only for AVs from men. Gal-3: galectin-3; IL: interleukin; CCL-2: C–C motif chemolike ligand 2; ICAM-1: Intercellular Adhesion Molecule 1; CD: cluster of differentiation. N = 108 AVs from men and N = 83 AVs from women. **Figure S3.** Gal-3 correlates with osteogenic markers only in men AVs. Positive correlations between Gal-3 and BMP-2 (A-B) in both men and women AVs. Positive correlations only in men AVs between Gal-3 and BMP-9 (C-D), osteopontin (E–F) and osteocalcin (G-H). Gal-3: galectin-3; BMP: bone morphogenetic protein. N = 108 AVs from men and N = 83 AVs from women. **Figure S4.** Gal-3 correlates with angiogenic markers only in men AVs. Positive correlations only in men AVs between Gal-3 and VEGF-A (A-B), FGF-7 (C-D) and VEGFR3 (E–F). Gal-3: galectin-3; VEGF: vascular endothelial growth factor; FGF-7: fibroblast growth factor-7; VEGFR: receptor of vascular endothelial growth factor. N = 108 AVs from men and N = 83 AVs from women.

**Additional file 2: Table S1.** Major resource table.

## Data Availability

The data that support the findings of this study are available from the corresponding author upon reasonable request. Mass-spectrometry data and search results files were deposited in the Proteome Xchange Consortium via the JPOST partner repository (https://repository.jpostdb.org) (57) with the identifier PXD042586 for ProteomeXchange and JPST002174 for jPOST (for reviewers: https://repository.jpostdb.org/preview/16863978726477857f0d462; Access key: 4749).
