# Supplementary figures and images for: Sex-specific role of galectin-3 in aortic stenosis
Source: Biol Sex Differ. 2023 Oct 24;14:72. doi: 10.1186/s13293-023-00556-1 (PMC10598900; doi:10.1186/s13293-023-00556-1)

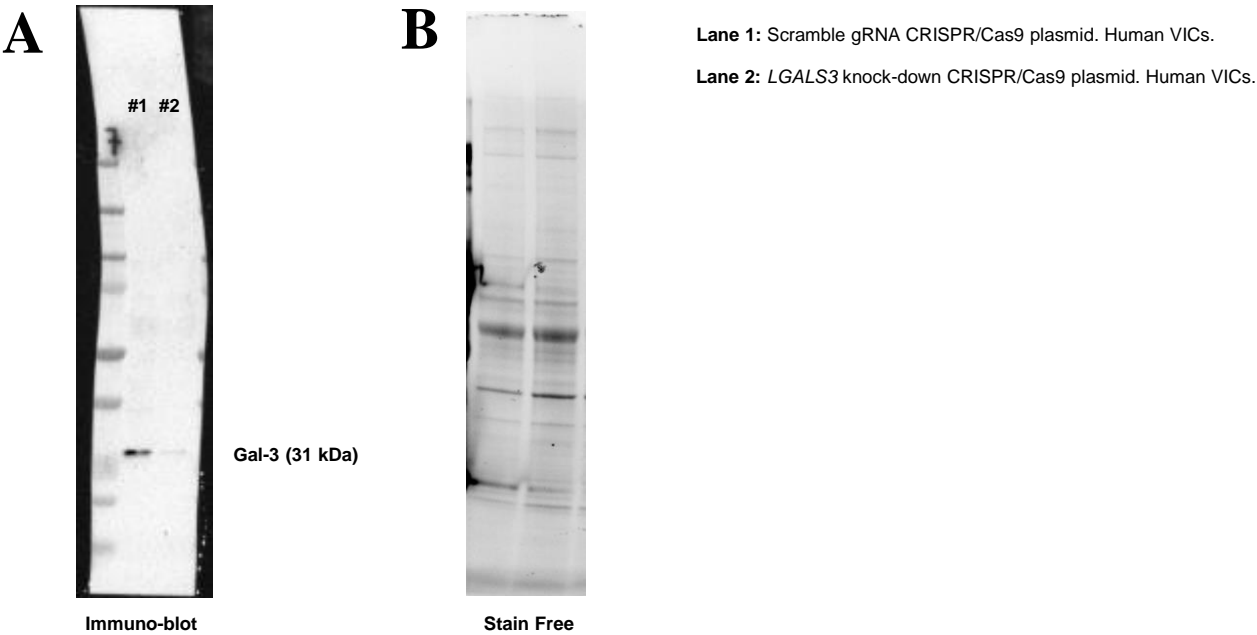

**A****Men**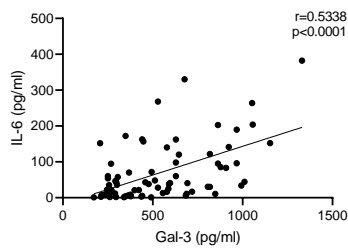**B****Women**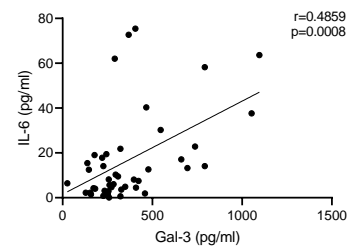**C**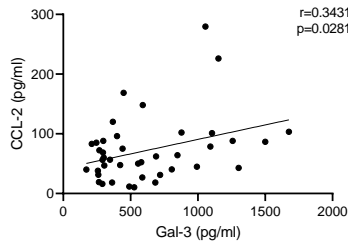**D**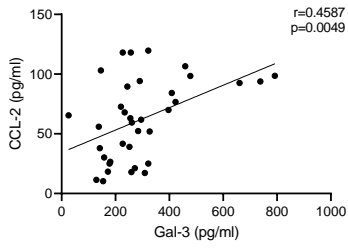**E**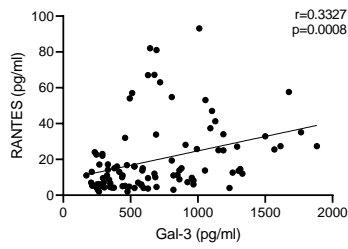**F**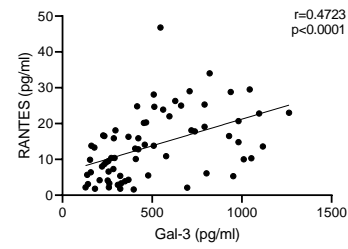**G**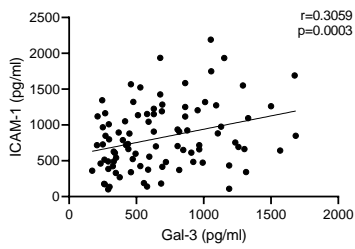**H**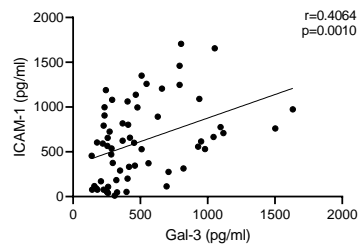**I**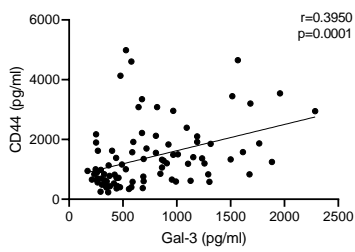**J**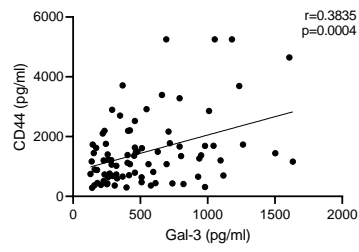**K**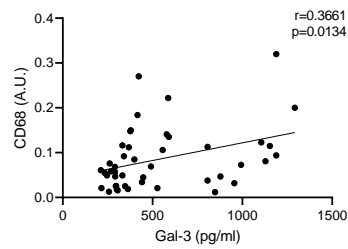**L**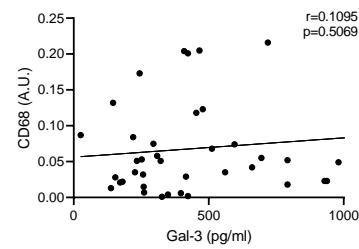**Figure S2**

**A****Men**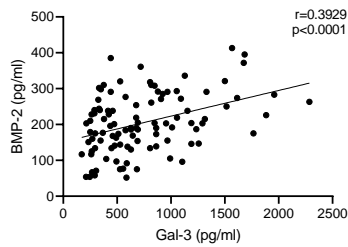**B****Women**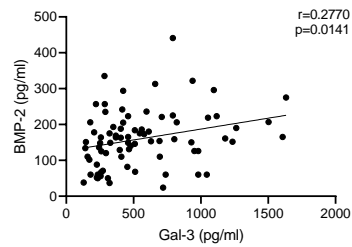**C**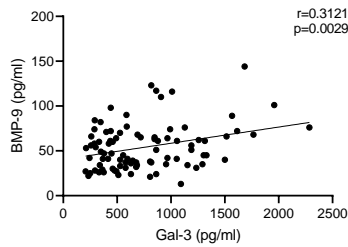**D**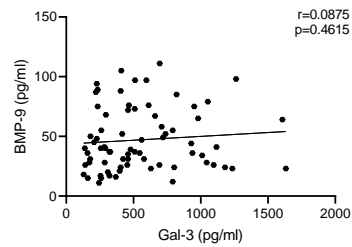**E**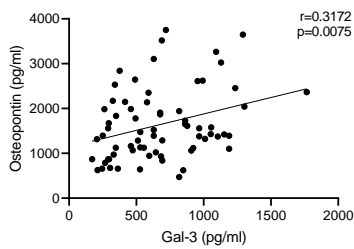**F**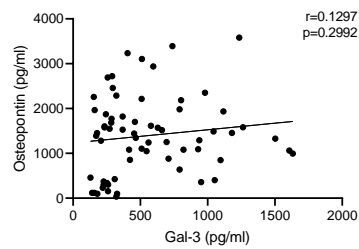**G**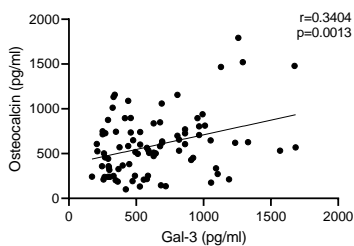**H**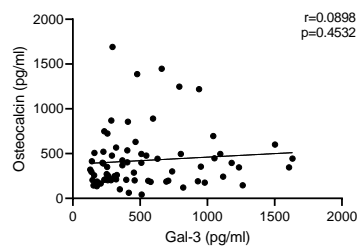**Figure S3**

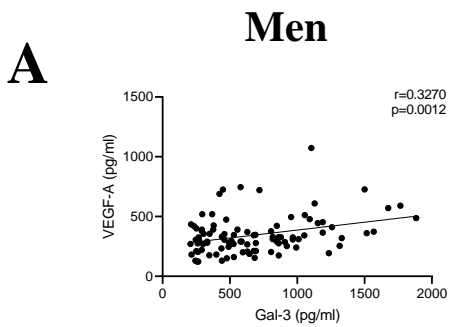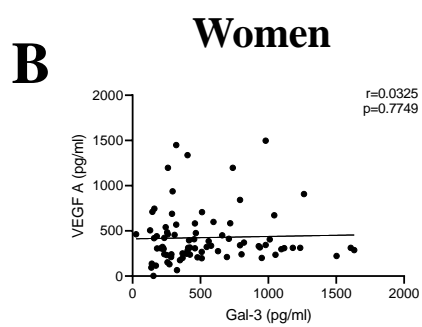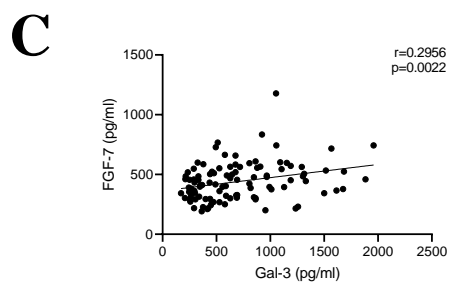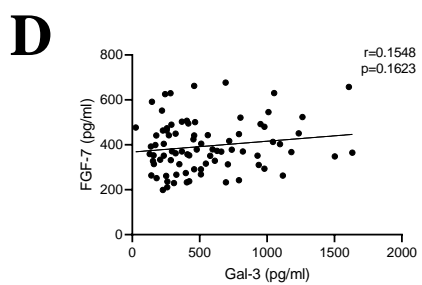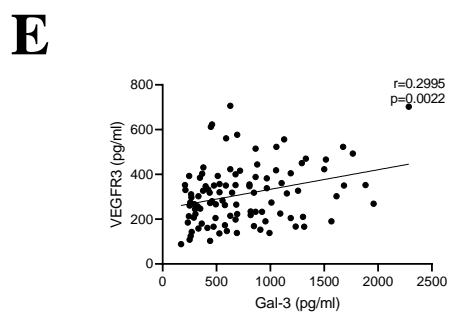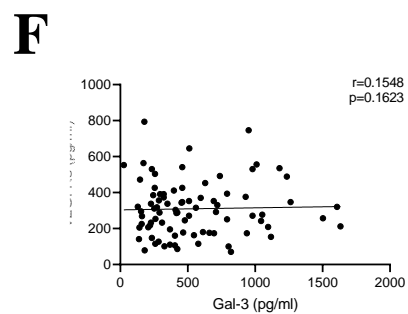

**Figure S4**

Supplement: Supplementary file 1 — Additional file 1: Figure S1. Gal-3 antibody validation in LGALS3-knockdown VICs. Immunoblot for Gal-3 commercial antibody in CRISPR/Cas9 edited VICs for scramble control (left) or LGALS3 gene knock-down (right) (A). Stain-free gel for the Gal-3 antibody validation immunoblot (B). Figure S2. Gal-3 correlates with inflammation markers both in men and women AVs. Positive correlations between Gal-3 and IL-6 (A-B), CCL-2 (C-D), RANTES (E–F), ICAM-1 (G-H), CD44 (I-J) for both men and women AVs. Positive correlation Gal-3-CD68 (K-L) only for AVs from men. Gal-3: galectin-3; IL: interleukin; CCL-2: C–C motif chemolike ligand 2; ICAM-1: Intercellular Adhesion Molecule 1; CD: cluster of differentiation. N = 108 AVs from men and N = 83 AVs from women. Figure S3. Gal-3 correlates with osteogenic markers only in men AVs. Positive correlations between Gal-3 and BMP-2 (A-B) in both men and women AVs. Positive correlations only in men AVs between Gal-3 and BMP-9 (C-D), osteopontin (E–F) and osteocalcin (G-H). Gal-3: galectin-3; BMP: bone morphogenetic protein. N = 108 AVs from men and N = 83 AVs from women. Figure S4. Gal-3 correlates with angiogenic markers only in men AVs. Positive correlations only in men AVs between Gal-3 and VEGF-A (A-B), FGF-7 (C-D) and VEGFR3 (E–F). Gal-3: galectin-3; VEGF: vascular endothelial growth factor; FGF-7: fibroblast growth factor-7; VEGFR: receptor of vascular endothelial growth factor. N = 108 AVs from men and N = 83 AVs from women. [file 13293_2023_556_MOESM1_ESM.pdf]
