# Supplementary material for: Sex-specific role of galectin-3 in aortic stenosis
Source: Biol Sex Differ. 2023 Oct 24;14:72. doi: 10.1186/s13293-023-00556-1 (PMC10598900; doi:10.1186/s13293-023-00556-1)
Supplement: Supplementary file 2 — Additional file 2: Table S1. Major resource table. [file 13293_2023_556_MOESM2_ESM.pdf]

Table S1. Major resource table

Antibodies

Target antigen	Vendor or Source	Catalog #	Working concentration	Persistent ID / URL
Gal-3	Santa Cruz Biotec.	sc-374253	0.16 µg/ml (IHQ), 2 µg/ml (WB)	https://www.scbt.com/es/p/galectin-3-antibody-h-5
α-SMA	Sigma-Aldrich	A7607	10 µg/ml	https://www.sigmaaldrich.com/ES/es/product/sigma/a7607
Vimentin	Santa Cruz Biotec.	sc-373717	2 µg/ml	https://www.scbt.com/es/p/vimentin-antibody-e-5
CD31	Santa Cruz Biotec.	sc-376764	2 µg/ml	https://www.scbt.com/p/pecam-1-antibody-h-3
VE-cadherin	Santa Cruz Biotec.	sc-9989	1.3 µg/ml	https://www.scbt.com/p/ve-cadherin-antibody-f-8
CD68	Abcam	Ab201340	1:100 (IHQ), 1:100 (WB)	https://www.abcam.com/cd68-antibody-c68684-ab201340.html
CD206	Santa Cruz Biotec.	sc-376108	2 µg/ml	https://www.scbt.com/p/cd206-antibody-d-1
CD45	Santa Cruz Biotec.	sc-28369	0.006 µg/ml	https://www.scbt.com/p/cd45-antibody-b-8
CD80	Santa Cruz Biotec.	sc-376012	4 µg/ml	https://www.scbt.com/p/b7-1-antibody-f-7
Osteopontin	Santa Cruz Biotec.	sc-21742	0.25 µg/ml	https://www.scbt.com/p/opn-antibody-akm2a1
Runx2	Sigma-Aldrich	AMAb90591	1 µg/ml	https://www.sigmaaldrich.com/ES/es/product/sigma/amab90591
Sox9	Sigma-Aldrich	AMAb90795	1 µg/ml (IHQ), 10 µg/ml (WB)	https://www.sigmaaldrich.com/ES/es/product/sigma/amab90795
BMP-2	Abcam	ab14933	1:150	https://www.abcam.com/bmp2-antibody-ab14933.html
VEGF-A	Santa Cruz Biotec.	sc-7269	0.2 µg/ml	https://www.scbt.com/p/vegf-antibody-c-1
VEGFR3	Santa Cruz Biotec.	sc-28297	2 µg/ml	https://www.scbt.com/p/flt-4-antibody-d-6

ELISA

Target antigen	Vendor or Source	Catalog #	Persistent ID / URL
Gal-3	R&D System	DY1154	https://www.rndsystems.com/products/human-galectin-3-duoset-elisa_dy1154
IL-6	R&D System	DY206	https://www.rndsystems.com/products/human-il-6-duoset-elisa_dy206
CCL2	R&D System	DY279	https://www.rndsystems.com/products/human-ccl2-mcp-1-duoset-elisa_dy279
Rantes	R&D System	DY278	https://www.rndsystems.com/products/human-ccl5-rantes-duoset-elisa_dy278
ICAM-1	R&D System	DY720	https://www.rndsystems.com/products/human-icam-1-cd54-duoset-elisa_dy720
CD44	R&D System	DY7045	https://www.rndsystems.com/products/human-cd44s-duoset-elisa_dy7045-05
VEGF-A	R&D System	DY293B	https://www.rndsystems.com/products/human-vegf-duoset-elisa_dy293b
VEGFR3	R&D System	DY349B	https://www.rndsystems.com/products/human-svegfr3-flt-4-duoset-elisa_dy349b-05
FGF-7	R&D System	DY251	https://www.rndsystems.com/products/human-kgf-fgf-7-duoset-elisa_dy251
Chemerin	R&D System	DY2324	https://www.rndsystems.com/products/human-chemerin-duoset-elisa_dy2324
BMP-2	R&D System	DY355	https://www.rndsystems.com/products/human-bmp-2-duoset-elisa_dy355
BMP-9	R&D System	DY314	https://www.rndsystems.com/products/human-bmp-4-duoset-elisa_dy314
Osteopontin	R&D System	DY1433	https://www.rndsystems.com/products/human-osteopontin-opn-duoset-elisa_dy1433
Osteocalcin	R&D System	DY1419	https://www.rndsystems.com/products/human-osteocalcin-duoset-elisa_dy1419-05
